# Immune Absence as a Proposed Framework for Delayed Relapse After Curative-Intent Treatment in Human Papillomavirus-Associated Cervical Cancer

**DOI:** 10.3390/cancers18162530

**Published:** 2026-08-07

**Authors:** Norihito Kamo, Shu Soeda, Tsuyoshi Honda, Keiya Fujimori

**Affiliations:** 1 Department of Obstetrics and Gynecology, Fukushima Medical University School of Medicine, Fukushima 960-1295, Japan; 2 Department of Regional Gynecologic Oncology, Fukushima Medical University School of Medicine, Fukushima 960-1295, Japan; 3 Department of Obstetrics and Gynecology, Iwaki City Medical Center, Iwaki 973-8555, Japan; 4 Department of Community Obstetrics and Gynecology Support, Fukushima Medical University School of Medicine, Fukushima 960-1295, Japan

**Keywords:** immune absence, late recurrence, cervical cancer, immune surveillance, minimal residual disease, circulating tumor DNA, T-cell receptor clonotypes

## Abstract

Delayed relapse after curative-intent treatment remains difficult to explain and predict in human papillomavirus (HPV)-associated cervical cancer, including after periods of sustained remission with undetectable circulating tumor DNA (ctDNA) or circulating HPV DNA. This limitation of tumor-centered surveillance demands the evaluation of immune parameters alongside tumor-derived biomarkers. We propose “immune absence” as a hypothesis-generating, operational framework referring to the sustained reduction or non-persistence of pre-specified tumor-reactive T-cell receptor (TCR) clonotypes in serial peripheral blood samples, obtained during minimal residual disease states in which antigen exposure may be limited or intermittent. We hypothesize that this longitudinal pattern may precede molecular or clinical evidence of relapse in a subset of patients and coexist with established mechanisms, such as tumor evolution, immune escape, T-cell dysfunction, and therapeutic resistance. Integrating serial ctDNA or circulating HPV DNA measurements with tumor-reactive TCR clonotype dynamics may provide complementary information on residual tumor burden and the persistence of circulating tumor-reactive T-cell responses. Peripheral blood TCR non-detection does not establish complete loss of anti-tumor immunity and may reflect compartmentalized immunity, clonal replacement, antigenic evolution, or assay limitations. Prospective longitudinal studies are required to establish the prognostic value of this framework before clinical use.

## Simple Summary

Some patients with human papillomavirus (HPV)-associated cervical cancer experience recurrence after apparently successful treatment, even when follow-up blood tests show no detectable circulating tumor DNA (ctDNA) or circulating HPV DNA. This perspective proposes “immune absence” as an operational framework referring to the sustained reduction or non-persistence of pre-specified tumor-reactive T-cell receptor (TCR) clonotypes in serial peripheral blood samples. This pattern may occur in settings where residual tumor antigen exposure is limited, but it does not indicate the complete loss of anti-tumor immunity and does not replace established mechanisms of relapse, including tumor evolution, immune escape, and T-cell dysfunction. Monitoring tumor-derived DNA biomarkers together with tumor-reactive TCR clonotypes may help characterize both residual tumor burden and the persistence of circulating tumor-reactive T-cell responses. Prospective studies are required before this framework can be used clinically.

## 1. Introduction

Late relapse after curative-intent treatment remains an important clinical challenge in human papillomavirus (HPV)-associated cervical cancer. Although many patients achieve apparent complete remission after surgery, chemoradiation, or combined-modality treatment, some subsequently develop recurrence after a clinically silent interval. This pattern is particularly difficult to interpret when conventional surveillance shows no radiographic evidence of disease and circulating tumor DNA (ctDNA) or circulating HPV DNA remains undetectable during follow-up [1,2,3,4,5,6,7]. Such cases suggest that tumor-derived biomarkers alone may not fully capture the biological state preceding relapse.

Molecular residual disease markers, including ctDNA and circulating HPV DNA, provide sensitive, tumor-centered measures of residual disease burden [1,2,3,4,5,6,7]. However, they primarily indicate whether tumor-derived nucleic acids are detectable in the circulation and do not directly assess the persistence of tumor-reactive immune responses. A biomarker-negative state may therefore represent true disease eradication, residual disease below the analytical detection threshold, low or intermittent DNA shedding, spatially confined disease, or a tumor–immune equilibrium not captured by circulating tumor-derived biomarkers.

Post-curative relapse may arise through overlapping mechanisms, including residual tumor burden, tumor evolution, immune escape, therapeutic resistance, and impaired anti-tumor immune function [8,9,10,11,12,13,14,15,16]. During minimal residual disease (MRD), antigen exposure may be limited or intermittent [2,6,7,17], potentially affecting the maintenance and peripheral detectability of antigen-specific T-cell populations [18,19,20,21]. These populations may undergo contraction, clonal replacement, redistribution, or functional alteration. Whether such longitudinal changes represent normal post-treatment immune remodeling or identify a biologically relevant pre-relapse state remains unresolved.

HPV-associated cervical cancer provides a tractable setting in which to investigate this question. Viral antigens offer relatively defined tumor-associated targets, while ctDNA or circulating HPV DNA provides a parallel measure of tumor-derived molecular disease [4,5,22,23,24,25]. Longitudinal assessment of these biomarkers together with pre-specified tumor-reactive T-cell receptor (TCR) clonotypes may therefore allow residual tumor burden and the persistence of circulating tumor-reactive T-cell responses to be examined concurrently.

Herein, we propose “immune absence” as a hypothesis-generating, operational framework referring to the sustained reduction or non-persistence of pre-specified tumor-reactive TCR clonotypes in serial peripheral blood samples during antigen-limited MRD states. This pattern does not imply the complete absence of anti-tumor immunity across tissues or immune compartments and may coexist with established mechanisms of relapse. The central question is whether longitudinal changes in these clonotypes are associated with subsequent molecular or clinical evidence of relapse in a subset of patients.

The present framework is focused on HPV-associated cervical cancer as a proof-of-concept setting for longitudinal tumor–immune monitoring. Its application to other gynecologic malignancies remains exploratory and will require disease-specific validation.

## 2. Central Hypothesis and Concept

We use the term “immune absence” to refer to the sustained reduction or non-persistence of pre-specified tumor-reactive T-cell receptor (TCR) clonotypes in serial peripheral blood samples during minimal residual disease (MRD) states, when antigen exposure may be limited or intermittent [9,10,18,19,20,21]. This pattern does not imply the complete absence of anti-tumor immunity across tissues or biological compartments; rather, it provides a basis for investigating changes in the persistence of circulating tumor-reactive T-cell responses.

Immune absence should be evaluated as a longitudinal pattern rather than an inference drawn from a single measurement. A negative peripheral blood TCR result may reflect sampling variation, low cellular input, insufficient sequencing depth, assay sensitivity, or transient immune-cell redistribution. Accordingly, serial analyses should assess sustained clonotype contraction, repeated non-detection, or replacement by alternative tumor-reactive clones. Tumor-reactive clonotypes should ideally be defined at baseline using paired tumor and blood analyses, viral antigen specificity, neoantigen prediction, or functional validation where feasible.

These longitudinal patterns may represent biologically distinct processes and should not be treated as interchangeable. Sustained contraction refers to a reproducible decrease in the abundance of a previously detectable clonotype, whereas repeated non-detection indicates that the clonotype remains below the assay detection threshold across consecutive samples. Clonal replacement describes the emergence of alternative tumor-reactive clonotypes despite the reduced persistence of those identified at baseline [15]. Distinguishing these patterns will require pre-specified analytical criteria that account for baseline abundance, sequencing depth, replicate consistency, and total T-cell input. Their biological significance may also differ depending on whether they occur in isolation or together with changes in tumor-derived biomarker detectability.

Following tumor debulking or definitive therapy, reduced or intermittent antigen exposure may affect the maintenance of antigen-specific T-cell populations [18,19,20,21]. Longitudinal changes in tumor-reactive clonotypes may therefore coexist with, or potentially precede, established mechanisms of post-curative relapse, including immune escape, T-cell dysfunction, tumor evolution, and therapeutic resistance. The proposed framework is intended to characterize a candidate immune–tumor state rather than replace these mechanisms.

Interpretation of reduced peripheral clonotype detectability must account for tissue-resident immunity, immune-cell redistribution, clonal replacement, antigenic evolution, and analytical variation [15,22,24,25,26,27,28]. Operational assessment should therefore consider baseline clonotype abundance, replicate consistency, sequencing depth, total T-cell input, and assay-specific detection limits, particularly during molecularly negative follow-up (Table 1).

The central testable prediction is that sustained changes in pre-specified tumor-reactive TCR clonotypes may precede molecular, clinical, or radiographic evidence of relapse in a subset of patients. This prediction should be evaluated by integrating serial ctDNA or circulating HPV DNA measurements with TCR sequencing, immune phenotyping, and clinically adjudicated relapse endpoints.

### 2.1. Relationship to Memory Maintenance, Immune Senescence, and Immune Surveillance Failure

Immune absence should be distinguished from impaired memory T-cell maintenance and generalized immune senescence. Impaired maintenance of antigen-specific memory T cells may contribute to the proposed immune-absence state in some settings where antigen exposure is limited; however, the two concepts are not synonymous. Memory maintenance refers to the long-term persistence, homeostatic renewal, and functional preservation of antigen-experienced T-cell populations [18,19,20,21], whereas immune absence refers to the sustained reduction or non-persistence of pre-specified tumor-reactive T-cell receptor (TCR) clonotypes in serial peripheral blood samples. Impaired memory maintenance may therefore represent one biological process contributing to this longitudinal pattern, but immune absence is defined by the observed clonotype trajectory rather than by a specific cellular mechanism.

Immune senescence, by contrast, encompasses broader changes in T-cell proliferative capacity, differentiation, repertoire diversity, and effector function, often associated with aging or chronic inflammation and potentially affecting multiple immune compartments [29,30]. These changes may influence the abundance, persistence, or functional competence of tumor-reactive T-cell populations. Nevertheless, generalized immune senescence is distinct from the immune-absence framework.

Immune surveillance failure should likewise be regarded as a functional outcome requiring direct investigation rather than inferred from peripheral blood non-detection alone. Reduced or non-persistent detection of circulating tumor-reactive TCR clonotypes may identify a candidate immune–tumor state associated with altered peripheral T-cell persistence, but it does not by itself establish loss of protective anti-tumor immunity or failure of immune control within tissues.

### 2.2. Disease-Specific Scope and Relevance

HPV-associated cervical cancer provides a tractable proof-of-concept setting for the immune-absence framework because defined viral antigens permit assessment of antigen-specific T-cell responses, while circulating HPV DNA provides a parallel tumor-derived biomarker for longitudinal monitoring [4,5,22,23,24,25]. This combination allows tumor burden and tumor-reactive immune persistence to be evaluated within the same disease-specific framework. In addition, tumor tissue and peripheral blood can be analyzed together to identify and track pre-specified tumor-reactive T-cell receptor (TCR) clonotypes over time. Studies of cervical cancer-associated T-cell repertoires and tissue-resident immune compartments further support the evaluation of both circulating and compartmentalized tumor-reactive immune responses [22,23,24,25].

Extension of this framework to ovarian, endometrial, vulvar, and other gynecologic cancers remains exploratory. These diseases differ in antigenic targets, immune microenvironments, tumor-derived DNA assay performance, treatment pathways, and relapse kinetics. Unlike HPV-associated cervical cancer, many of these tumors lack shared viral antigens, and identification of tumor-reactive TCR clonotypes may therefore depend more heavily on tumor sequencing, neoantigen prediction, functional assays, or paired tumor and blood analyses. Differences in tissue distribution and patterns of recurrence may also influence the relationship between peripheral clonotype detectability and tissue-based immune control. Accordingly, the present framework should be interpreted primarily within HPV-associated cervical cancer, and application to other gynecologic malignancies will require disease-specific analytical criteria and prospective validation.

## 3. Biological and Clinical Rationale

Current evidence provides a biological rationale for examining changes in the persistence of circulating tumor-reactive T-cell responses during post-curative minimal residual disease (MRD), although it does not establish immune absence as a causal mechanism of relapse. This rationale has two related components: antigen availability may influence the maintenance of tumor-reactive T-cell populations, and tumor-derived biomarkers may not fully capture the immune context preceding relapse.

**Figure 1:**
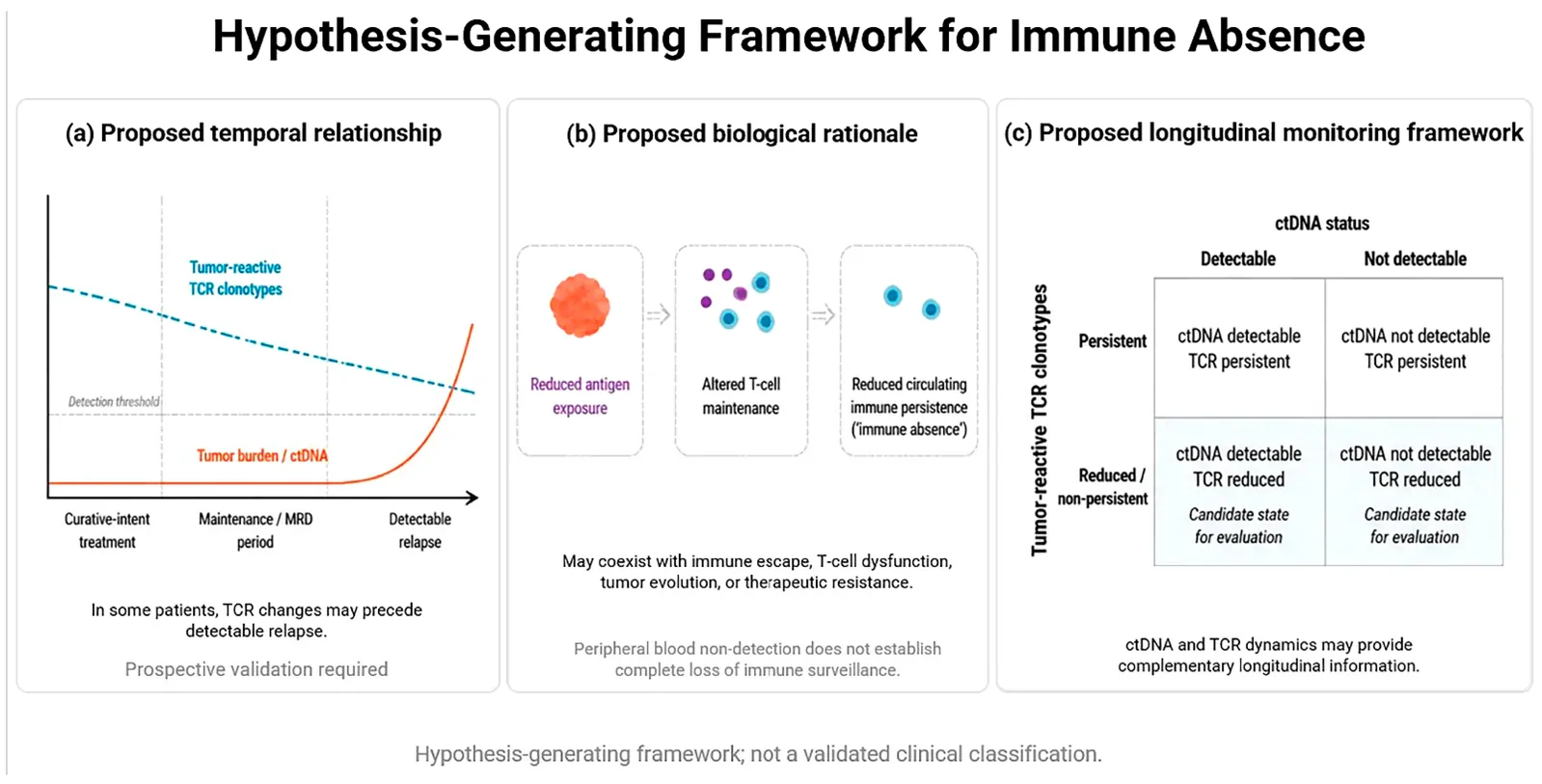
Hypothetical framework of antigen-limited immune persistence and post-curative relapse. (**a**) Proposed temporal relationship between pre-specified tumor-reactive T-cell receptor (TCR) clonotype dynamics and recurrence. In a subset of patients, sustained reduction or non-persistence of these clonotypes in serial peripheral blood samples may precede molecular, clinical, or radiographic evidence of relapse, while circulating tumor DNA (ctDNA) or circulating HPV DNA remains below the analytical detection threshold. (**b**) Proposed biological rationale. Following tumor debulking or definitive therapy, limited or intermittent antigen exposure may affect the maintenance and peripheral detectability of tumor-reactive T-cell populations. During minimal residual disease (MRD), sustained reduction, or non-persistence of pre-specified tumor-reactive TCR clonotypes is operationally termed immune absence. (**c**) Proposed framework for longitudinal tumor-derived biomarker and TCR monitoring. Patients may be characterized according to the detectability of ctDNA or circulating HPV DNA and the persistence of pre-specified tumor-reactive TCR clonotypes. A biomarker-negative state accompanied by sustained reduction or non-persistence of these clonotypes represents the candidate immune–tumor pattern central to the immune-absence framework. Abbreviations: ctDNA, circulating tumor DNA; HPV, human papillomavirus; MRD, minimal residual disease; TCR, T-cell receptor. This figure presents a hypothesis-generating framework for prospective evaluation and does not define a validated clinical risk classification.

Following tumor debulking or definitive therapy, residual tumor burden and antigen exposure may become low or intermittent [2,6,7,17]. Reduced antigenic stimulation may alter the maintenance and peripheral detectability of antigen-specific T-cell populations [18,19,20,21]. Preclinical models of micrometastatic disease also support the coexistence of limited residual tumor burden and altered immune dynamics [17]. In this setting, sustained reduction or non-persistence of pre-specified tumor-reactive T-cell receptor (TCR) clonotypes in serial peripheral blood samples may represent a measurable feature of an antigen-limited immune state (Figure 1b).

The relationship between antigen availability and tumor-reactive T-cell persistence is unlikely to be uniform across clonotypes or memory T-cell subsets. Some antigen-experienced T-cell populations may persist through homeostatic mechanisms despite limited ongoing antigen exposure, whereas others may depend more strongly on intermittent antigenic stimulation or supportive cellular niches [18,19,20,21]. Reduced peripheral detectability may therefore reflect differences in clonal maintenance, anatomical localization, or antigen dependence rather than a single biological process. Tissue-resident populations may remain functionally relevant even when corresponding clonotypes become less detectable in peripheral blood, while newly emerging clonotypes may replace those identified at baseline [15,23,28]. Integration of longitudinal clonotype tracking with phenotypic, functional, and tissue-based analyses will be necessary to distinguish among these possibilities.

Tumor-derived biomarkers, including circulating tumor DNA (ctDNA) or circulating HPV DNA, provide information on residual tumor burden but do not directly assess the persistence of tumor-reactive T-cell responses. A biomarker-negative state may reflect true disease eradication, residual disease below the analytical detection threshold, limited DNA shedding, or assay-related constraints. Longitudinal assessment of pre-specified tumor-reactive TCR clonotypes may therefore provide complementary information. The central hypothesis is that sustained changes in these clonotypes may precede molecular, clinical, or radiographic evidence of relapse in a subset of patients while tumor-derived DNA remains undetectable (Figure 1a).

Reduced peripheral clonotype detectability does not establish complete loss of anti-tumor immunity and may reflect tissue-resident immunity, immune-cell redistribution, clonal replacement, antigenic evolution, or analytical variation [15,22,23,24,25,28]. The prognostic value of this pattern should therefore be evaluated in prospective, disease-specific studies comparing longitudinal TCR trajectories with ctDNA or circulating HPV DNA and clinically adjudicated relapse endpoints.

## 4. Clinical and Methodological Implications

The proposed framework is intended to complement established post-curative surveillance based on clinical assessment, imaging, and tumor-derived biomarkers, including circulating tumor DNA (ctDNA) or circulating HPV DNA [1,2,3,4,5]. These approaches assess residual tumor burden but do not directly measure the persistence of circulating tumor-reactive T-cell responses. Integrating tumor-derived biomarkers with longitudinal T-cell receptor (TCR) monitoring may therefore provide a broader view of post-treatment tumor–immune status.

Conceptually, combined tumor-derived DNA and TCR monitoring may define four candidate post-curative patterns: biomarker-positive/TCR-persistent, biomarker-positive/TCR-reduced, biomarker-negative/TCR-persistent, and biomarker-negative/TCR-reduced. Here, TCR-reduced refers to sustained reduction or non-persistence of pre-specified tumor-reactive TCR clonotypes across serial peripheral blood samples. The immune-absence framework primarily concerns the biomarker-negative/TCR-reduced pattern, in which tumor-derived DNA remains below the analytical detection threshold while circulating tumor-reactive clonotypes show a sustained change (Figure 1c). These patterns are proposed for prospective comparison and do not represent established clinical risk groups.

The biological and prognostic significance of these patterns is likely to depend more on longitudinal trajectory than on a single measurement. A reproducible transition from biomarker-negative/TCR-persistent to biomarker-negative/TCR-reduced status may be more informative than an isolated low TCR measurement or persistently low clonotype abundance from baseline. Conversely, reappearance or expansion of previously reduced clonotypes may reflect renewed antigen exposure, redistribution into the circulation, or changes in immune activation rather than restoration of effective tumor control. Transitions involving biomarker positivity may also differ according to whether tumor-reactive clonotypes remain detectable, contract, or are replaced by alternative clones. Analyses should therefore evaluate within-patient state transitions and their temporal relationship to molecular, clinical, or radiographic evidence of relapse.

Interpretation of these trajectories will require consideration of both analytical and biological variability. Baseline identification of pre-specified tumor-reactive TCR clonotypes, standardized sampling intervals, defined analytical thresholds, and prespecified handling of false-negative and false-positive results will be important. Relevant factors include baseline clonotype abundance, magnitude and duration of change, replicate consistency, sequencing depth, total T-cell input, and assay-specific variability. Complementary immune phenotyping and paired tissue and blood analyses may help distinguish reduced peripheral detectability from tissue compartmentalization, clonal replacement, phenotypic change, or evolving antigen specificity.

The combined framework may also help define priorities for future surveillance research. For example, studies could compare whether biomarker/TCR transitions provide greater prognostic information than absolute measurements at individual time points and whether particular trajectories are associated with different relapse sites, intervals, or treatment histories. Such analyses may clarify whether longitudinal immune monitoring contributes information that is independent of, or biologically complementary to, established clinicopathologic factors and tumor-derived biomarkers.

At present, the principal value of this framework lies in supporting structured longitudinal investigations of post-curative tumor–immune trajectories. Clinical application will require a prospective demonstration that integrated tumor-derived biomarker and TCR monitoring provides reproducible prognostic value beyond established surveillance approaches.

## 5. Testable Predictions and Validation Strategy

The central testable prediction is that longitudinal integration of tumor-derived biomarkers and T-cell receptor (TCR) clonotype dynamics may provide prognostic information beyond isolated measurements of either component. Specifically, sustained reduction or non-persistence of pre-specified tumor-reactive TCR clonotypes in serial peripheral blood samples may precede molecular, clinical, or radiographic evidence of recurrence in a subset of patients, including during periods when circulating tumor DNA (ctDNA) or circulating HPV DNA remains undetectable.

**Figure 2:**
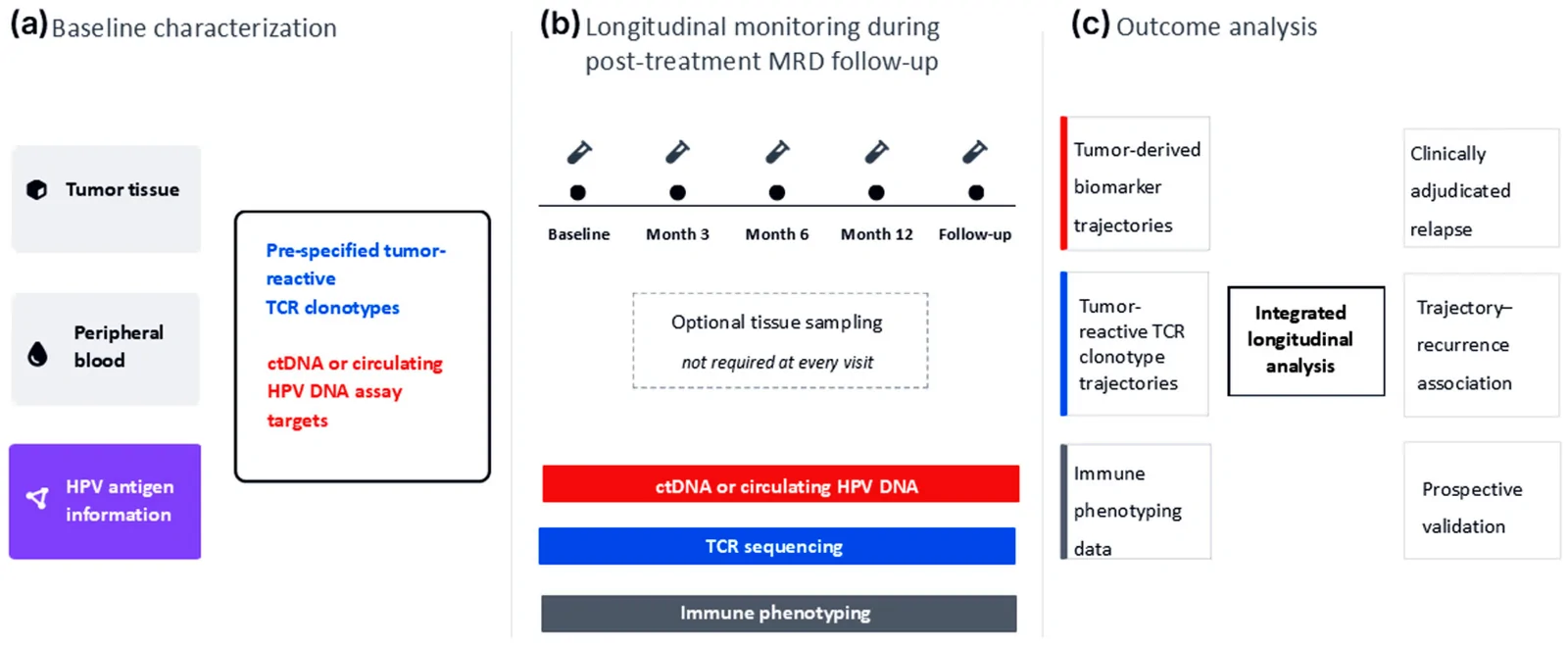
(**a**) Baseline characterization after curative-intent treatment. Tumor tissue, peripheral blood, and available disease-specific antigen information are used to identify pre-specified tumor-reactive T-cell receptor (TCR) clonotypes and establish circulating tumor DNA (ctDNA) or circulating HPV DNA assay targets. (**b**) Longitudinal monitoring during post-treatment minimal residual disease (MRD) follow-up. Serial blood samples are collected at pre-specified intervals for ctDNA or circulating HPV DNA measurement, TCR sequencing, and immune phenotyping. Where clinically feasible, tissue sampling is included to assess tissue-resident immunity and tumor antigen evolution. (**c**) Outcome analysis. Longitudinal tumor-derived biomarker and TCR trajectories are compared with clinically adjudicated relapse endpoints to determine whether sustained reduction or non-persistence of pre-specified tumor-reactive TCR clonotypes is reproducibly associated with subsequent recurrence. This figure presents a proposed validation strategy for prospective evaluation and does not represent a clinical management algorithm.

This prediction should be evaluated in prospective longitudinal studies using clinically adjudicated relapse endpoints (Figure 2). At baseline, paired tumor and blood samples should be used to identify pre-specified tumor-reactive TCR clonotypes and establish ctDNA or circulating HPV DNA assay targets. Antigen specificity or functional tumor reactivity should be confirmed where feasible (Figure 2a) [22,24,25].

During post-treatment follow-up, serial blood samples should be collected at prospectively defined intervals for ctDNA or circulating HPV DNA measurement, TCR sequencing, and immune phenotyping (Figure 2b). Sampling schedules should be established before outcome assessment. Where clinically feasible, tissue sampling may provide additional information on tissue-resident immunity, clonal redistribution, and tumor antigen evolution.

Longitudinal tumor-derived biomarker and TCR trajectories should then be compared with clinically adjudicated relapse endpoints (Figure 2c). Analyses should determine whether sustained changes in pre-specified tumor-reactive TCR clonotypes provide prognostic information beyond tumor-derived biomarkers and conventional clinicopathologic factors. Relevant covariates may include disease stage, nodal status, treatment modality, HPV genotype, baseline tumor burden, lymphocyte count, and assay-specific detection limits.

Predefined endpoints may include sustained clonotype contraction, repeated non-detection, transitions between tumor-derived biomarker/TCR patterns, and the interval between clonotype change and subsequent relapse. Time-dependent or landmark-based approaches may help evaluate the relationship between serial biomarker changes and clinical outcomes. Analytical thresholds, sampling schedules, and endpoint definitions should be prespecified and subsequently evaluated in an independent validation cohort.

HPV-associated cervical cancer provides a practical proof-of-concept setting because defined viral antigens and circulating HPV DNA permit parallel longitudinal assessment of residual tumor burden and circulating tumor-reactive T-cell responses [4,5,22,24,25,31,32,33]. Clinical application will require prospective demonstration of reproducible prognostic value beyond established surveillance approaches.

### Limitations

Identifying and longitudinally tracking pre-specified tumor-reactive T-cell receptor (TCR) clonotypes remains technically challenging, particularly during antigen-limited minimal residual disease (MRD), when relevant T-cell populations may be scarce [15,22,24,25,26,27]. Assay sensitivity, sequencing depth, analytical thresholds, cellular input, and inter-sample variability may affect estimates of clonotype persistence. Tumor antigen heterogeneity and temporal evolution may also limit the ability of a fixed set of clonotypes to represent tumor-reactive immune responses over time [11,13,15].

Peripheral blood profiling captures only part of the anti-tumor immune response. Reduced or non-persistent detection of tumor-reactive TCR clonotypes may reflect tissue-resident immunity, immune-cell redistribution, clonal replacement, phenotypic change, or technical non-detection rather than loss of protective immunity [23,28]. Paired tissue and blood analyses, together with complementary immune phenotyping, will therefore be important for distinguishing changes in the persistence of circulating tumor-reactive T-cell responses from compartmentalized or evolving immune responses.

The temporal relationship among TCR clonotype dynamics, tumor-derived biomarker detectability, and relapse remains undefined and may vary according to patient characteristics and treatment. Prospective studies are required to determine whether longitudinal TCR trajectories provide reproducible prognostic information beyond ctDNA or circulating HPV DNA and established clinicopathologic factors.

## 6. Conclusions

Immune absence is proposed as a framework referring to the sustained reduction or non-persistence of pre-specified tumor-reactive T-cell receptor (TCR) clonotypes in serial peripheral blood samples during antigen-limited minimal residual disease states. This pattern may provide an immune dimension complementary to tumor-derived biomarkers, including circulating tumor DNA (ctDNA) or circulating HPV DNA.

The central question is whether longitudinal tumor-derived biomarker and TCR trajectories are reproducibly associated with subsequent relapse. Addressing this question will require prospective, disease-specific studies using standardized clonotype identification, serial sampling, complementary immune phenotyping, and clinically adjudicated relapse endpoints. Clinical application will depend on demonstrating prognostic value beyond established surveillance approaches.

## Figures and Tables

**Table 1:** Proposed operational distinctions among post-curative immune–tumor states.

Term	Definition	Key Characteristic
Immune escape	Tumor persistence or progression associated with reduced immune recognition or effector function, including antigen loss, HLA downregulation, or immunosuppressive mechanisms.	Tumor adaptation limits effective immune control.
Immune exhaustion	Functional impairment of tumor-reactive T cells associated with sustained antigen exposure and exhaustion-related phenotypic or transcriptional features.	T cells persist but have reduced effector capacity.
Immune absence	Sustained reduction or non-persistence of pre-specified tumor-reactive TCR clonotypes across serial peripheral blood samples during molecular remission.	A proposed marker of reduced circulating immune persistence during antigen-limited minimal residual disease states.
Maintenance/MRD context	Post-curative period without clinically detectable disease, during which residual tumor burden and antigen exposure may be low or intermittent.	A clinical context for longitudinal tumor–immune monitoring.

TCR, T-cell receptor; HLA, human leukocyte antigen. Note: These categories are not mutually exclusive and are not intended as validated clinical classifications. Peripheral blood non-detection of TCR clonotypes does not establish complete loss of immune surveillance and may reflect tissue redistribution, tissue-resident immunity, clonal replacement, antigenic evolution, or assay limitations.

## Data Availability

No new data were created or analyzed in this study. Data sharing is not applicable to this article.

## Abbreviations

The following abbreviations are used in this manuscript:

ctDNA	Circulating tumor DNA
DNA	Deoxyribonucleic acid
HLA	Human leukocyte antigen
HPV	Human papillomavirus
MRD	Minimal residual disease
TCR	T-cell receptor
